# Prescribing practices for presumptive TB among private general practitioners in South Africa: a cross-sectional, standardised patient study

**DOI:** 10.1136/bmjgh-2021-007456

**Published:** 2022-01-18

**Authors:** Angela Salomon, Jody Boffa, Sizulu Moyo, Jeremiah Chikovore, Giorgia Sulis, Benjamin Daniels, Ada Kwan, Tsatsawani Mkhombo, Sarah Wu, Madhukar Pai, Amrita Daftary

**Affiliations:** 1 School of Medicine, Queen's University, Kingston, Ontario, Canada; 2 McGill International TB Centre, McGill University, Montréal, Quebec, Canada; 3 Division of Biostatistics and Epidemiology, Stellenbosch University, Stellenbosch, South Africa; 4 Centre for Rural Health, University of KwaZulu-Natal, Durban, South Africa; 5 Human and Social Capabilities Programme, Human Sciences Research Council, Cape Town, South Africa; 6 School of Public Health and Family Medicine, University of Cape Town, Cape Town, South Africa; 7 School of Population and Global Health, McGill University, Montreal, Québec, Canada; 8 McCourt School of Public Policy, Georgetown University, Washington, DC, USA; 9 Division of Pulmonary and Critical Care Medicine, University of California School of Medicine, San Francisco, California, USA; 10 Human and Social Capabilities Programme, Human Sciences Research Council, Durban, South Africa; 11 Mailman School of Public Health, Columbia University, New York, New York, USA; 12 School of Global Health & Dahdaleh Institute of Global Health Research, York University, Toronto, Ontario, Canada; 13 Centre for the AIDS Programme of Research, Durban, KwaZulu-Natal, South Africa

**Keywords:** tuberculosis, epidemiology, health services research, screening, HIV

## Abstract

**Introduction:**

Medicine prescribing practices are integral to quality of care for leading infectious diseases such as tuberculosis (TB). We describe prescribing practices in South Africa’s private health sector, where an estimated third of people with TB symptoms first seek care.

**Methods:**

Sixteen standardised patients (SPs) presented one of three cases during unannounced visits to private general practitioners (GPs) in Durban and Cape Town: TB symptoms, HIV-positive; TB symptoms, a positive molecular test for TB, HIV-negative; and TB symptoms, history of incomplete TB treatment, HIV-positive. Prescribing practices were recorded in standardised exit interviews and analysed based on their potential to contribute to negative outcomes, including increased healthcare expenditures, antibiotic overuse or misuse, and TB diagnostic delay. Factors associated with antibiotic use were assessed using Poisson regression with a robust variance estimator.

**Results:**

Between August 2018 and July 2019, 511 SP visits were completed with 212 GPs. In 88.5% (95% CI 85.2% to 91.1%) of visits, at least one medicine (median 3) was dispensed or prescribed and most (93%) were directly dispensed. Antibiotics, which can contribute to TB diagnostic delay, were the most common medicine (76.5%, 95% CI 71.7% to 80.7% of all visits). A majority (86.1%, 95% CI 82.9% to 88.5%) belonged to the WHO Access group; fluoroquinolones made up 8.8% (95% CI 6.3% to 12.3%). Factors associated with antibiotic use included if the SP was asked to follow-up if symptoms persisted (RR 1.14, 95% CI 1.04 to 1.25) and if the SP presented as HIV-positive (RR 1.11, 95% CI 1.01 to 1.23). An injection was offered in 31.9% (95% CI 27.0% to 37.2%) of visits; 92% were unexplained. Most (61.8%, 95% CI 60.2% to 63.3%) medicines were not listed on the South African Primary Healthcare Essential Medicines List.

**Conclusion:**

Prescribing practices among private GPs for persons presenting with TB-like symptoms in South Africa raise concern about inappropriate antimicrobial use, private healthcare costs and TB diagnostic delay.

**Key questions**

**What is already known?**

- South Africa ranks among the highest tuberculosis (TB) and HIV-associated TB burden countries globally and has a thriving private healthcare sector, where up to a third of people with TB symptoms first seek care.
- Overuse, underuse or misuse of medicines can contribute to adverse drug events, diagnostic delay, antibiotic resistance and growing healthcare expenditures.
- Little is known about prescribing practices in the private sector and how they contribute to quality of TB and HIV care.

**What are the new findings?**

- When presented with an standardised patient (SP) reporting typical TB symptoms and HIV on probing, private general practitioners (GPs) provided at least one medicine 98.0% of the time, including an antibiotic 89.6% of the time.
- Most antibiotics (86.1%) were from the ‘Access’ category of the WHO Access, Watch and Reserve framework, indicating lower risk of antibiotic resistance. However, prescription of antibiotics can mask TB symptoms and lead to TB diagnostic delay. The fluoroquinolone class of antibiotics can also contribute to resistance to second-line TB treatment drugs and made up 8.8% of all antibiotic use in this sector.
- Factors associated with antibiotic use included if there was diagnostic uncertainty, if the SP was asked to return if symptoms persisted, if the SP presented as HIV-positive, and if the GP asked <3 TB-related questions on history.

**What do the new findings imply?**

- Prescribing practices among private South African GPs presented with TB-like symptoms raise concerns about inappropriate antimicrobial use, higher healthcare costs and TB diagnostic delay.
- These practices may reflect lack of access to point-of-care testing, diagnostic uncertainty and the need to strengthen private–public referral mechanisms.

## Background

Medicines play a crucial role in the delivery of primary healthcare. Their rational use, defined by WHO as ‘appropriate to (a patient’s) clinical needs, in doses that meet their own individual requirements, for an adequate period of time and at the lowest cost to them and their community’,^1^ contributes to disease prevention, alleviation and treatment. Their inappropriate use (ie, overuse, underuse or misuse), however, can compromise optimal disease management and trigger avertable complications such as adverse drug events, drug dependence, antimicrobial resistance and patient and health system expenditures. This is especially problematic for severe diseases such as tuberculosis (TB), where inappropriate medicine use can also mask symptoms crucial for diagnosis and lead to delays in the testing, diagnosis and confirmation of TB.^2–5^ Investigation into medicine prescribing practices is thus essential to evaluate overall quality of care in primary care settings.

In South Africa, TB is a leading cause of morbidity and mortality.^6^ Lower respiratory symptoms suggestive of pulmonary TB, among other differential diagnoses, are among the most common clinical presentations in primary care.^7^ Studies in other low-income and middle-income countries demonstrate high rates of inappropriate medicine use, particularly antibiotic use, in primary care settings.^8 9^ In South Africa’s public sector, where most patients with presumptive TB are seen, the rate of inappropriate antibiotic use is estimated to be around 8%.^10^ However, a third of such patients are estimated to first present to the private sector,^11^ where medicine prescribing practices in response to presentations of TB are understudied. These practices may be further complicated in the South African context by the high underlying prevalence of HIV (19% in the general adult population and 58% among people with TB),^12^ which demands patients receive concurrent and integrated attention for not only one but two potentially serious conditions.

Evaluating medicine prescribing practices for TB and HIV-associated TB in the private sector can inform and strengthen national responses to TB, especially given that the private sector has been implicated in TB diagnostic delay.^13–15^ Recently, our team published results of a standardised patient (SP) study that evaluated quality of care for presumptive TB and HIV-associated TB through various indicators including clinical examination, testing and referral practices in the cities of Durban and Cape Town. Over 511 simulated patient interactions with 212 private general practitioners (GPs), we found TB and HIV were ideally managed only 43% and 41% of the time, respectively.^16^ In this paper, we describe a subanalysis of medicine prescribing practices among participating GPs to provide further evidence on quality of care gaps and opportunities for enhancing management of TB and HIV-associated TB in South Africa’s private sector.

## Methods

The study used SP methodology, whereby locally recruited and extensively trained staff portray a standardised case presentation during a clinical encounter, which is then documented to measure quality of care.^17^ The SP methodology provides a measure for provider practice that is not subject to typical biases and confounders (eg, patient mix and patient sorting) present in other quality of care methods such as clinical observation, clinician surveys or vignette-based assessments. It has been validated in a number of disease states, including TB.^17 18^

### Data collection

The study was based in South Africa, where the annual incidence rate of TB is 615 per 100 000.^12^ Study communities included urban and peri-urban areas of two cities, Durban and Cape Town. Recruitment and data collection methods, as well as the SP training protocol, have been previously published.^16^ Briefly, in each study community, eight SPs were recruited and trained in one of three presentations (table 1). SPs portraying case 1 or ‘typical TB’ presented with classic TB symptoms and were HIV-positive and antiretroviral therapy (ART)-naïve on probing. SPs portraying case 2 or ‘confirmed TB’ presented with typical TB symptoms and a positive laboratory report for the detection of Mycobacterium tuberculosis (via Xpert MTB/Rif assay, Cepheid, Sunnyvale, California, USA) and were HIV-negative on probing. SPs portraying case 3 or ‘previous TB’ presented with typical TB symptoms and a history of incomplete TB treatment, indicating they were HIV-positive and ART-naïve on probing. Training followed an extensive protocol borrowing on previous SP work in TB and tailored to the South African context.^17^ SPs were assessed and confirmed to be in apparent good health to mitigate confounding during clinical encounters.

**Table 1:** SP case scenarios

Standardised patient case	Opening statement	Relevant history (if prompted)	Ideal TB management strategy*
Case 1: ‘Basic TB’	I have a cough and am feeling hot, and it’s not getting better	Cough duration 2 weeks, experiencing loss of weight/appetite and night sweats, known HIV+, not on ART	Offered/sent for any TB test or referred to public sector for any reason
Case 2: ‘Confirmed TB’	I have a cough that is not getting better. I have been to a clinic back home and they gave me some tablets and took my spit	Carrying GeneXpert pos/Rif inconclusive laboratory report. Cough duration 3 weeks, experiencing loss of weight/appetite and night sweats, HIV—at last test 1 year ago	
Case 3: ‘Previous TB’	I am suffering from a bad cough. About a year ago I had got tablets in the hospital, and it had got better. But now again I’m having this cough	Cough duration 2 weeks, experiencing loss of weight/appetite and night sweats, diagnosed and treated with TB last year at which time took 3–4 months TB treatment, known HIV+, not on ART	

*South African TB guidelines were used as a reference for ideal TB management, which was defined as a verbal or written (1) recommendation for any TB or HIV-related test or (2) referral to the public sector for any reason.

ART, antiretroviral therapy; SPs, standardised patients; TB, tuberculosis.

All GPs registered with the Health Professions Council of South Africa and working within independent private clinics in urban and periurban wards of the two study communities that met the following criteria were included: (1) ≥20% of ward with annual household income <40 000 South African rand (ZAR; approximately US$3000), (2) ≥1000 black Africans by subplace (to reflect the local population and minimise SP detection), (3) presence of >2 private GPs and (4) within reasonable distance to a public clinic and accessible by public transportation. GPs practising exclusively at private hospitals or providing care to specialised populations (eg, paediatrics, obstetrics) were excluded. Consenting GPs (n=212) received up to three unannounced SPs: the majority (202; 95.3%) received case 1 and a random sub-sample subsequently received cases 2 and/or 3 in succession to reduce any priming effect. One hundred and one providers (47.6%) received all three cases. Immediately following each visit, SPs completed a facilitated interaction survey to record clinical practices, and submitted any artefacts, including medications and prescription notes, given to them by the GP for documentation. To minimise SP detection, each SP portrayed a single case, all SPs presented as walk-in cash-paying patients, no GP working within the same practice received the same case more than once, and a minimum of 2 weeks passed before any GP received a second (or third) case.

### Analysis

This study follows the same analytical approach to interaction surveys as Boffa *et al*,^16^ where the South African TB guidelines—that do not recommend antibiotic treatment for TB or HIV-associated TB without a TB test—were used as a reference to evaluate ideal TB and HIV management.^19^ All interaction artefacts (medicines, sputum cups, prescriptions, referral letters, sick notes) were submitted to research staff, anonymized, labelled, photographed and filed according to interaction number. An artefact survey was then completed by trained research staff and double-entered into SurveyCTO (Dobility, Cambridge, Massachusetts, USA). For all prescriptions and medicine products, the following data were documented: active ingredient/s, name (generic or brand), formulation (tablet, syrup, nasal spray, inhaler, cream or other), expiry date, number of pills dispensed and instructions for use, drug classification (drawing on the Anatomical Therapeutic Chemical classification system),^8^ and whether the medicine was on the South Africa Primary Healthcare Essential Medicines List (EML).^20^ Antibiotics were also classified according to the 2019 WHO Access, Watch and Reserve framework based on potential for selecting resistance.^21^ ‘Access’ antibiotics have activity against a wide range of commonly encountered susceptible pathogens and are thus recommended as the first line of treatment for several infections; ‘Watch’ antibiotics are broad-spectrum molecules that should be employed with greater caution; ‘Reserve’ antibiotics are last-resort drugs that should be kept for treatment of confirmed infections due to multidrug-resistant and extremely drug-resistant organisms; and ‘Discouraged’ antibiotics include fixed-dose combinations (FDC) that lack evidence-based indications for use.

We explored prescribing practices using descriptive statistics (proportions, 95% CIs using bootstrapped estimators of variance, medians and IQR as appropriate) and compared practices between case presentations and study site (Durban vs Cape Town). We examined factors associated with antibiotic use by fitting a series of bivariate Poisson regressions with a robust variance estimator, each adjusted for case presentation and study site because of their potential to contribute to antimicrobial resistance and TB diagnostic delay.^2–5^ However, as TB treatment initiation and management generally occurs in the public sector through the South African national TB programme, we did not analyse TB treatment initiation as an outcome of interest for private GPs. All provider demographics (sex, years in practice, place of training) are included in the bivariate analyses. SPs were considered comparable across SP–provider interactions; hence, we did not adjust for SP-specific demographic variables. All statistical analyses were conducted using Stata V.15.1 (StataCorp).

### Patient and public involvement

This study examines quality of healthcare, in particular quality of medication prescribing practices. The study used SP actors, and participants included physicians. Patients were not recruited or involved in study implementation. Physicians practising in the study communities were invited to attend a free seminar in which study findings were shared and where they could earn continuing medical education credits.

## Results

Between August 2018 and July 2019, 511 interactions (case 1=202, case 2=157, case 3=152) were completed with 212 consenting GPs (Durban=96, Cape Town=116). There was a higher proportion of female GPs and those practising in suburbs in Cape Town versus Durban. Other GP characteristics did not significantly differ between SP cases or study sites (table 2). GP participation rate was 57%. A flow chart depicting consent patterns is included in online supplemental 1.

10.1136/bmjgh-2021-007456.supp1 Supplementary data

**Table 2:** Provider characteristics by study site

	Overall (n=511)	Durban (n=220)	Cape Town (n=291)
Provider gender, n (%)*			
Male	375 (73.4)	189 (85.9)	186 (63.9)
Female	136 (26.6)	31 (14.1)	105 (36.1)
Years in practice, median (IQR)	25.5 (15–35.5)	25 (15–33)	26 (15–35.5)
Location of training, n (%)			
South African Institution	430 (84.3)	178 (81.3)	252 (86.6)
International Institution	80 (15.7)	41 (18.7)	39 (13.4)
Daily patient load, median (IQR)	26 (15–30)	25 (15–33)	20 (15–30)
Consult fee in ZAR†, median (IQR)	321 (280–380)	300 (260–350)	350 (300–398)
Area, n (%)*			
City	105 (20.6)	98 (44.6)	7 (2.4)
Township	119 (23.3)	95 (43.2)	24 (8.3)
Suburb	287 (56.2)	27 (12.3)	260 (89.4)

*P value through χ^2^ test of statistical significance <0.001.

†1 ZAR roughly equivalent to US$0.066.

### General patterns of medicines prescribed

A total of 1576 medicines were entered into the artefact surveys. At least one medicine was provided (prescribed or directly dispensed) in 452 of 511 interactions (88.5%, 95% CI 85.2% to 91.1%), with a median of 3 (IQR 2–4) medicines per interaction (table 3). The vast majority (92.8%, 95% CI 91.3% to 94.1%) of medicines were dispensed at the point of care (POC) in the sampled clinics; the remainder (7.2%, 95% CI 5.9% to 8.7%) were provided via prescription (table 4). Among medicines directly dispensed, 22 (1.5%, 95% CI 0.9% to 2.4%) were unlabelled (across 22 interactions), 3 (0.2%, 95% CI 0.0% to 0.5%) were expired (across three interactions) and 625 (46.5%, 95% CI 40.4% to 45.1%) had no visible expiry date (across 145 interactions).

**Table 3:** Prescribing practices by Interaction and SP case presentation

	Overall		Case 1		Case 2		Case 3	
	N	% (95% CI)	N	% (95% CI)	N	% (95% CI)	N	% (95% CI)
All interactions	511	–	202	–	157	–	152	–
No of medicines, median (IQR)	3 (2–4)		4 (3–4)		2 (0–3)		3 (2–4)	
At least one medicine	452	88.5 (85.2 to 91.1)	198	98.0 (95.0 to 99.2)	113	72.0 (65.6 to 77.6)	141	92.8 (88.6 to 95.5)
Any antibiotic	391	76.5 (71.7 to 80.7)	181	89.6 (84.8 to 93.0)	87	55.4 (47.7 to 62.9)	123	80.9 (72.7 to 87.1)
Asked about fever or took temperature	229	58.6 (54.0 to 63.0)	113	62.4 (55.8 to 68.6)	45	51.7 (42.8 to 60.6)	71	57.7 (47.9 to 70.0)
Auscultated lungs	367	93.9 (91.4 to 95.6)	174	96.1 (92.3 to 98.1)	78	89.7 (81.5 to 94.4)	115	93.5 (87.6 to 96.7)
Counselled on finishing antibiotic course	174	44.5 (39.2 to 50.0)	78	43.1 (36.1 to 50.3)	42	48.3 (38.7 to 58.0)	54	43.9 (36.9 to 51.1)
Concurrent Ideal TB Management*	223	57.0 (51.6 to 62.2)	75	41.4 (34.5 to 48.7)	66	75.9 (66.1 to 83.5)	82	66.7 (57.2 to 75.0)
Offered injection	163	31.9 (27.0 to 37.2)	83	41.1 (34.6 to 47.9)	29	18.5 (14.0 to 23.9)	51	33.6 (26.1 to 41.9)
Unexplained	150	92.0 (89.0 to 94.3)	77	92.8 (86.2 to 96.3)	24	82.8 (66.5 to 92.1)	49	96.1 (88.8 to 98.7)
‘Influenza/cough’	8	4.9 (2.8 to 8.5)	5	6.0 (2.8 to 12.5)	3	10.3 (3.1 to 2.9)	0	–
Antibiotic	4	2.5 (1.0 to 6.0)	1	1.2 (0.2 to 8.2)	2	6.9 (1.9 to 2.2)	1	2.0 (0.2 to 15.0)
Vitamin BCO	1	0.6 (0.2 to 2.3)	0	–	0	–	1	
Amount paid by SP, median (range)	330 (0–580)		345 (100–580)		340 (0–500)		320 (0–550)	
Medicines dispensed (n=421)	340 (30–580)		340 (150–580)		350 (30–500)		320 (150–550)	
No medicines dispensed (n=90)	320 (0–480)		350 (100–450)		310 (0–480)		300 (0–480)	

*Offered/sent for any TB test or referred to public sector for any reason.

BCO, B Complex; SP, standardised patient; TB, tuberculosis.

**Table 4:** Prescribing practices by individual medicine and SP case presentation

	Overall		Case 1		Case 2		Case 3	
	N	% (95% CI)	N	% (95% CI)	N	% (95% CI)	N	% (95% CI)
All medicines	1576	–	741	–	342	–	493	–
Medicine class								
Antibiotic	435	27.6 (25.2 to 30.1)	201	27.1 (69.8 to 75.7)	91	26.6 (22.2 to 31.5)	143	29.0 (25.0 to 33.3)
Cough remedy	327	20.7 (19.0 to 22.6)	142	19.1 (16.4 to 22.3)	84	24.6 (20.3 to 29.4)	101	20.5 (17.2 to 24.2)
Analgesic/antipyretic	203	12.9 (11.4 to 14.5)	96	13.0 (10.5 to 15.9)	47	13.7 (10.7 to 17.5)	60	12.2 (9.8 to 15.1)
Cold/influenza combination	170	10.8 (9.4 to 12.3)	82	11.(8.8 to 13.8)	37	10.8 (7.6 to 15.2)	51	10.3 (7.7 to 13.8)
Steroid	98	6.2 (5.2 to 7.5)	53	7.1 (5.4 to 9.3)	14	4.1 (2.4 to 6.9)	31	6.3 (4.7 to 8.3)
Vitamin/supplement	94	6.0 (5.0 to 7.1)	36	4.9 (3.5 to 6.8)	23	6.7 (4.2 to 10.6)	35	7.1 (5.1 to 9.8)
Antihistamine	88	5.6 (4.6 to 6.7)	47	6.3 (4.6 to 8.7)	16	4.7 (2.8 to 7.7)	25	5.1 (3.5 to 7.3)
Bronchodilator	62	3.9 (3.0 to 5.2)	27	3.6 (2.4 to 5.5)	9	2.6 (1.4 to 5.0)	26	5.3 (3.7 to 7.5)
NSAID	56	3.6 (2.9 to 4.4)	31	4.2 (3.0 to 5.8)	11	3.2 (1.8 to 5.8)	14	2.8 (1.6 to 4.9)
Herbal	9	0.6 (0.3 to 1.1)	3	0.4 (0.1 to 1.4)	4	1.2 (0.4 to 3.4)	2	0.4 (0.1 to 1.4)
Other	8	0.5 (0.2 to 1.1)	5	0.7 (0.3 to 1.4)	2	0.5 (0.1 to 2.4)	1	0.2 (0.0 to 2.0)
Formulation								
Tablets (pills)	1181	74.9 (73.0 to 76.7)	565	76.2 (73.9 to 78.4)	249	72.8 (66.5 to 78.3)	367	74.4 (71.1 to 77.5)
Syrups	387	24.6 (22.8 to 26.5)	171	23.1 (20.9 to 25.4)	93	27.1 (21.7 to 33.5)	123	24.9 (22.0 to 28.1)
Nasal spray	5	0.3 (0.2 to 0.6)	3	0.4 (0.2 to 0.9)	0	–	2	0.4 (0.0 to 1.7)
Oral inhaler	1	0.1 (0.0 to 0.3)	0	–	0	–	1	0.2 (0.1 to 0.7)
Cream	1	0.1 (0.0 to 0.2)	1	0.1 (0.0 to 0.6)	0	–	0	–
Other*	1	0.1 (0.0 to 0.2)	1	0.1 (0.0 to 1.3)	0	–	0	–
Delivery								
Prescribed	113	7.2 (5.9 to 8.7)	61	8.2 (6.6 to 10.2)	19	5.6 (3.6 to 8.4)	33	6.7 (4.8 to 9.3)
Dispensed	1463	92.8 (91.3 to 94.1)	680	91.8 (89.8 to 93.4)	323	94.4 (01.6 to 96.4)	460	93.3 (90.7 to 95.2)
Expired†								
Yes	3	0.2 (0.0 to 0.5)	1	0.1 (.0 to 1.7)	1	0.3 (0.0 to 1.7)	1	0.2 (0.0 to 1.0)
No	835	57.1 (54.6 to 59.5)	396	58.2 (54.9 to 61.5)	176	54.5 (49.7 to 59.2)	263	57.2 (52.6 to 61.6)
Unknown	625	42.7 (40.4 to 45.1)	283	41.6 (38.4 to 44.9)	146	45.2 (40.5 to 50.0)	196	42.6 (38.1 to 47.2)
Unlabelled†	22	1.5 (0.9 to 2.4)	17	2.5 (1.6 to 4.0)	3	0.9 (0.2 to 3.4)	2	0.4 (0.1 to 1.9)
All active ingredients	3485	–	1600	–	804	–	1081	–
South African EML								
Yes	1332	38.2 (36.6 to 40.0)	628	39.3 (37.4 to 41.1)	283	35.2 (31.7 to 38.9)	421	38.9 (35.9 to 42.0)
No	2153	61.8 (60.2 to 63.3)	972	60.8 (58.9 to 62.6)	521	64.8 (61.1 to 68.3)	660	61.2 (58.0 to 64.1)

*Ear drops.

†Among medicines directly dispensed (n=1463).

EML, Essential Medicines List; NSAID, Non-Steroidal Anti-Inflammatory Drug; SP, standardised patient.

**Figure 1:**
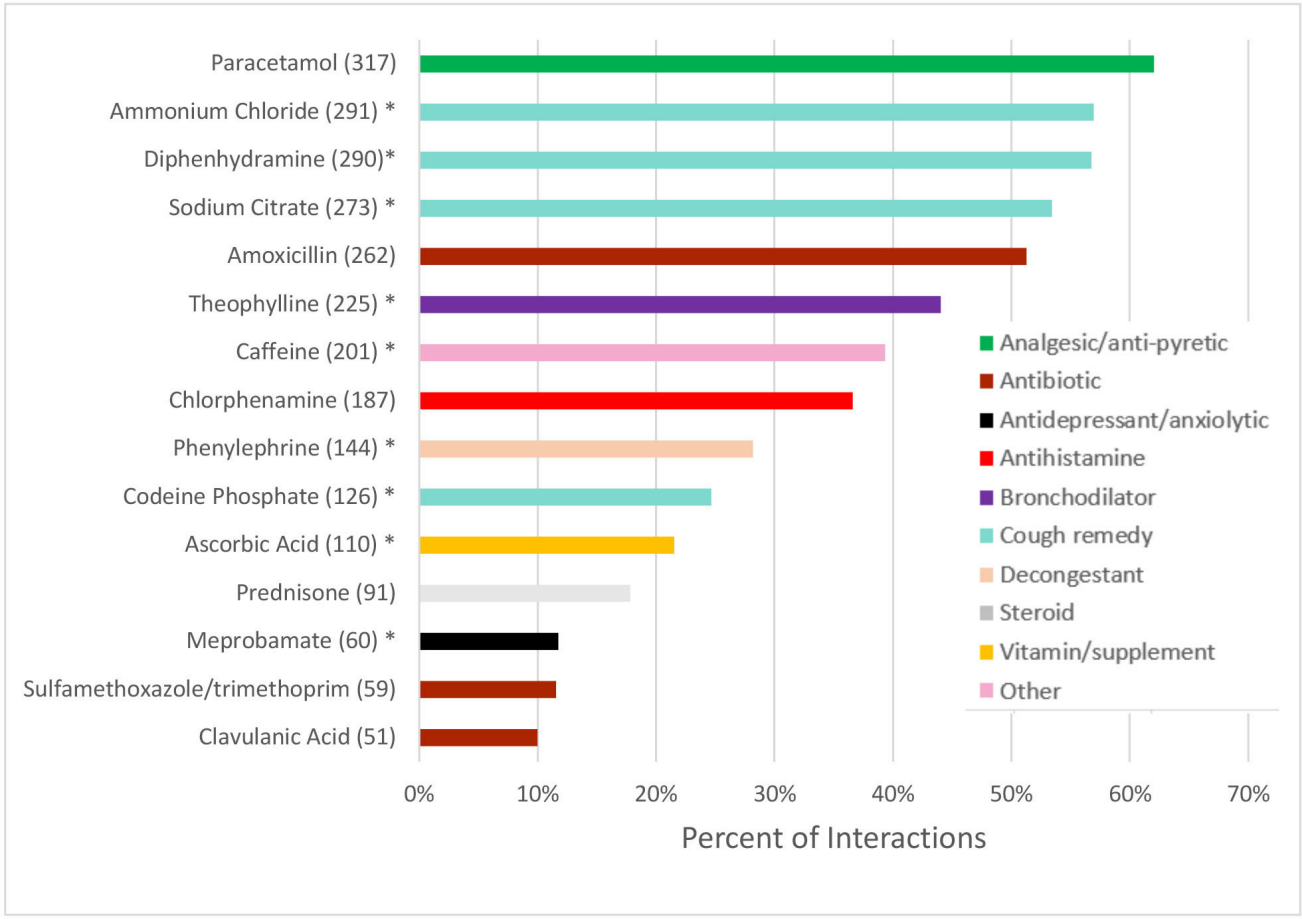
Most commonly prescribed/dispensed active ingredients. The number in brackets is the number of interactions in which that active ingredient was recorded. *Indicates an ingredient not listed on the South African Primary Healthcare Essential Medicines list.

The most common medicines were antibiotics (27.6% of all medicines) provided in 391 of 511 (76.5%) interactions. This was followed by cough remedies (20.7% of all medicines), analgesics/antipyretics such as paracetamol and paracetamol/codeine combinations (12.9% of all medicines), and cold medicine combinations of paracetamol plus phenylephrine or ephedrine (10.8% of all medicines). The most common formulations were tablets (n=1181, 74.9%) and syrups (n=387, 24.6%), with only one inhaler prescribed (a bronchodilator). The most common active ingredients were paracetamol, cough remedies (ammonium chloride, diphenhydramine and sodium citrate), amoxicillin and theophylline (figure 1); for a list of all active ingredients see online supplemental 2. Although no antiretrovirals were provided, SPs were referred to the public sector or another GP for HIV management and/or ART in 18.4% of interactions involving an HIV-positive case presentation (12.4% of case 1% and 26.3% of case 3). An injectable medication was offered in 163 interactions (31.9%, 95% CI 27.0% to 37.2%) (table 3). The majority of injections (92.0%, 95% CI 89.0% to 94.3%) were not explained to the SP, with the remainder described as ‘for influenza/cough’ (4.9%, 95% CI 2.8% to 8.5%), antibiotics (2.5%, 95% CI 1.0% to 6.0%) or a vitamin B complex (0.6%, 95% CI 0.2% to 2.3%). All injections were declined by the SP in line with their training.

The majority of medicines (61.8%, 95% CI 60.2% to 63.3%) were not listed on the South African Primary Healthcare EML^20^ (table 4). Ingredients not on the EML included those commonly in cough syrups (eg, ammonium chloride, dextromethorphan), bronchodilators (eg, theophylline), non-steroidal anti-inflammatories (eg, diclofenac, indomethacin), analgesics (eg, codeine, oxycodone) and antihistamines (eg, (des)loratadine, levocetirizine). See online supplemental 2 for a full list of concordance of active ingredients with the EML.

**Figure 2:**
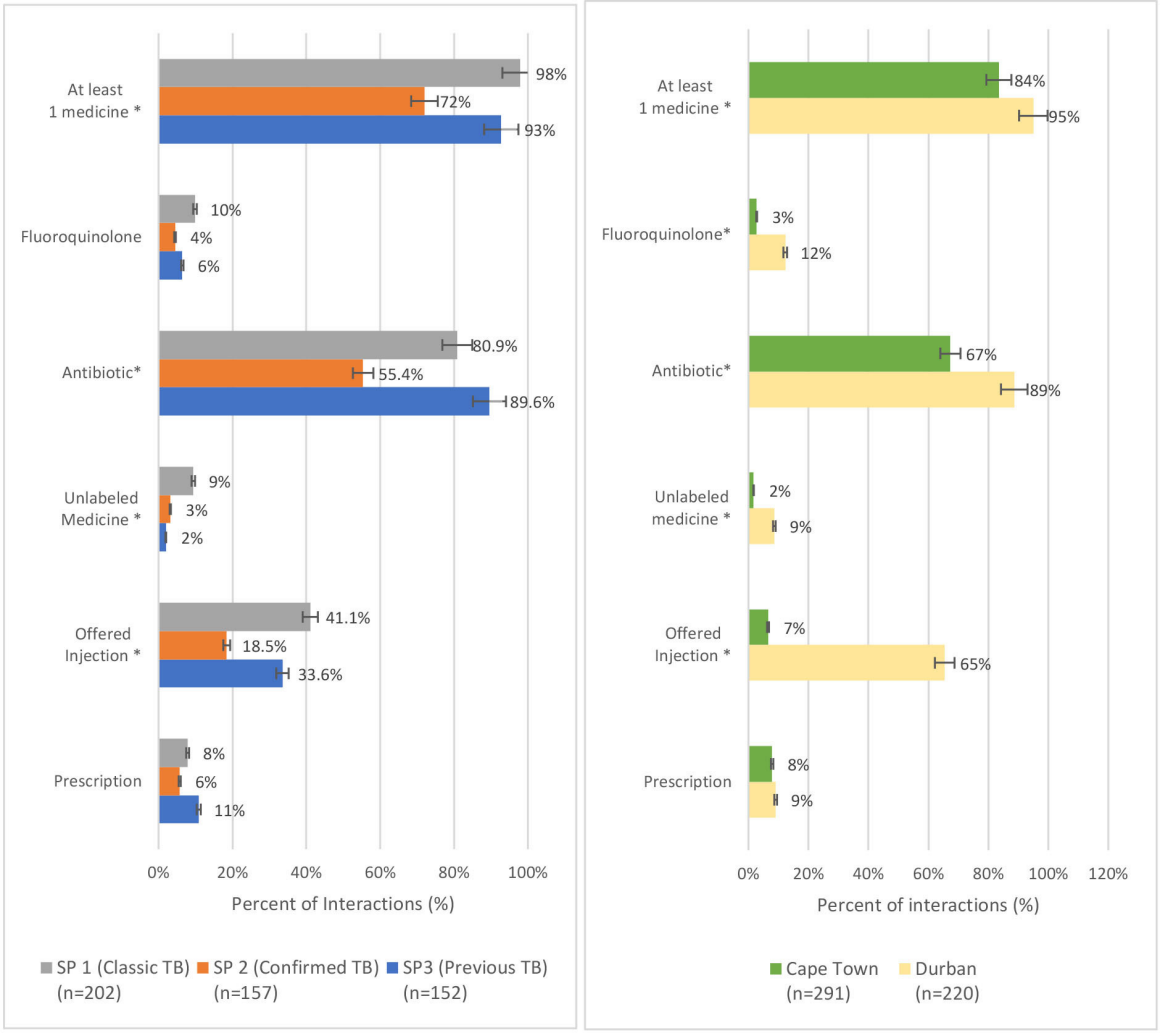
Prescription practices by case and study community. *Significantly different, p<0.05. SPs, standardised patients; TB, tuberculosis.

Patterns of medicines prescribed varied significantly by case and study site (figure 2). For example, significantly more interactions involving either ‘typical TB’ (case 1) or ‘previous TB’ (case 3) resulted in any medicine (Relative risk (RR) 1.3, 95% CI 1.2 to 1.5) or offer of an injection (RR 2.1, 95% CI 1.4 to 2.9) compared with ‘confirmed TB’ (case 2). Similarly, significantly more interactions in Durban resulted in provision of any medicine (RR 1.1, 95% CI 1.1 to 1.2), an unlabelled medicine (RR 5.2, 95% CI 1.9 to 13.3), or offer of an injection (RR 10.0, 95% CI 6.4 to 15.6) compared with those in Cape Town. There were no significant differences between case presentation or study site in the likelihood of prescribing versus directly dispensing a medicine.

### Antibiotic prescribing practices

Of 435 antibiotics provided, penicillins (amoxicillin and amoxicillin/clavulanic acid) were the most common, making up 64.0% (95% CI 59.2% to 68.6%), followed by trimethoprim-sulfamethoxazole (13.6%, 95% CI 10.7% to 17.0%) and fluoroquinolones (moxifloxacin, levofloxacin, ciprofloxacin) (8.8%, 95% CI 6.3% to 12.3%) (table 5, figure 1). The majority of antibiotics (86.1%, 95% CI 81.7% to 89.5%) were from the ‘Access’ group, 56 (13.0%, 95% CI 9.6% to 17.4%) were from the ‘watch’ group, and 1 (0.2%, 95% CI 0.0% to 1.9%) was a ‘Discouraged’ (FDC, ie, amoxicillin/flucloxacillin) (table 5). No antibiotics were given from the ‘Reserve’ group. Anti-TB drugs (isoniazid alone, or FDCs of isoniazid, rifampicin, pyrazinamide and ethambutol) were prescribed in three interactions (0.7% of all antibiotics, 95% CI 0.2 to 2.2), all involving the ‘confirmed TB’ presentation (case 2). Among 391 interactions where an antibiotic was provided, only 57.0% (95% CI 52.1% to 61.9%) had concurrent ‘Ideal TB Management’ (tables 1 and 3), and in only 44.5% (95% CI 39.2% to 50.0%) the SP was counselled on the importance of completing the antibiotic course. SPs were determined to be HIV-positive based on history taking in 32.8% of interactions where sulfamethoxazole/trimethoprim (also known as co-trimoxazole) was provided.

**Table 5:** Antibiotic-specific practices by individual medicine and SP case presentation

	Overall		Case 1		Case 2		Case 3	
	N	% (95% CI)	N	% (95% CI)	N	% (95% CI)	N	% (95% CI)
Any antibiotic	435	–	201	–	91	–	143	–
AWaRE classification								
Access	374	86.1 (82.9 to 88.5)	174	87.6 (83.0 to 91.0)	76	83.5 (76.4 to 88.8)	121	85.3 (77.0 to 91.0)
Watch	56	12.8 (10.2 to 16.0)	24	12.1 (8.8 to 16.0)	11	12.1 (7.3 to 19.3)	21	14.7 (9.0 to 23.0))
Reserve	0	–	0	–	0	–	0	–
Discouraged*	1	0.2 (0.0 to 2.0)	1	0.5 (0.1 to 1.8)	0	–	0	–
Other†	4	0.9 (0.3 to 2.4)	0	–	4	4.4 (1.3 to 14.0)	0	–
ATC classification								
Penicillin	275	63.2 (58.7 to 67.5)	138	68.7 (61.6 to 75.0)	50	54.9 (45.8 to 63.8)	87	60.8 (52.8 to 68.3)
Sulfonamide‡	59	13.6 (10.7 to 17.0)	23	11.4 (8.6 to 15.0)	11	12.1 (6.9 to 20.2)	25	17.5 (12.2 to 24.4)
Fluoroquinolone	38	8.8 (6.3 to 12.3)	15	7.5 (4.2 to 13.1)	7	7.8 (4.1 to 14.2)	16	11.3 (7.2 to 17.2)
Tetracycline	26	6.0 (4.6 to 7.8)	9	4.5 (2.3 to 8.6)	10	11.0 (6.5 to 17.9)	7	4.9 (2.3 to 9.9)
Macrolide	16	3.7 (2.5 to 5.5)	7	3.5 (1.5 to 8.0)	4	4.4 (1.6 to 11.9)	5	3.5 (1.5 to 7.8)
Cephalosporin	10	2.3 (1.1 to 4.7)	6	3.0 (1.5 to 6.1)	3	3.3 (1.5 to 7.2)	1	0.7 (0.1 to 4.4)
Imidazole§	6	1.4 (0.6 to 3.3)	2	1.0 (0.3 to 3.3)	2	2.2 (0.4 to 11.0)	2	1.4 (0.4 to 5.5)
Antimycobacterial	3	0.7 (0.2 to 2.2)	0	–	3	3.3 (1.7 to 9.1)	0	–
Combinations*¶	1	0.2 (0.0 to 1.5)	1	0.5 (0.2 to 1.6)	0	–	0	–
Unknown	1	0.2 (0.0 to 1.8)	0	–	1	1.1 (0.3 to 3.7)	0	–

*Amoxicillin/flucloxacillin fixed-dose combination.

†Anti-TB treatment (n=3), unknown antibiotic (n=1).

‡Sulfamethoxazole/trimethoprim (co-trimoxazole).

§Metronidazole.

¶Does not include antimycobacterial combinations.

ATC, Anatomical Therapeutic Chemical; AWaRE, Access, Watch and Reserve; SP, standardised patients; TB, tuberculosis.

**Figure 3:**
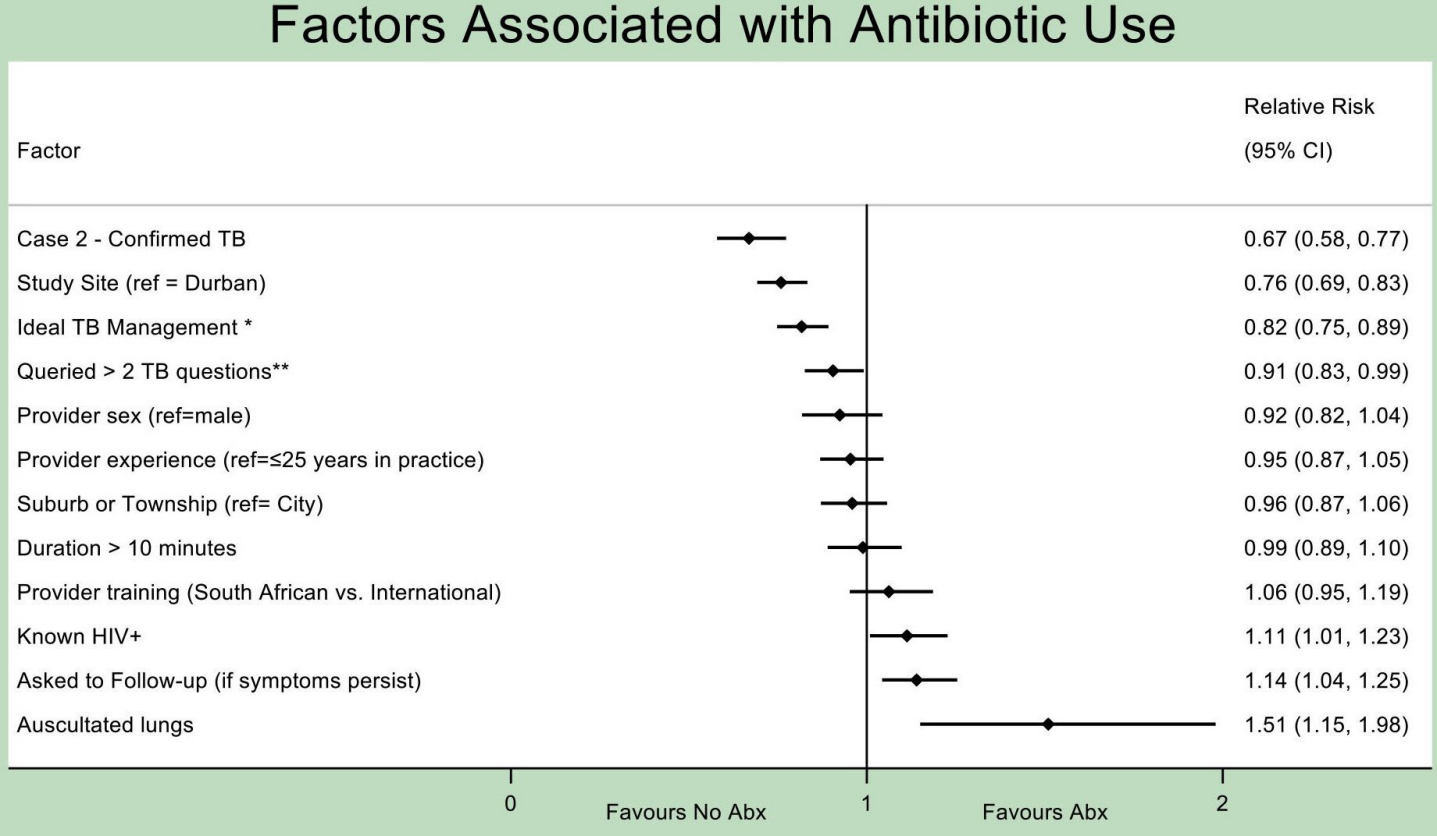
Factors associated with antibiotic dispensing. All analyses adjusted for SP case and study community. *Offered/sent for any TB test or referred to public sector for any reason. **Duration of cough, presence of fever, night sweats, loss of weight or appetite. SPs, standardised patients; TB, tuberculosis.

Factors associated with use of any antibiotic included performance of lung auscultation (RR 1.51, 95% CI 1.15 to 1.98), request for a follow-up visit if symptoms persist (RR 1.14, 95% CI 1.04 to 1.25), or determination of HIV-positivity through history taking (specific to cases 1 and 3, RR 1.11, 95% CI 1.01 to 1.23) (figure 3). Factors associated with non-use of an antibiotic included presentation of a ‘confirmed TB’ case (case 2) (RR 0.67, 95% CI 0.58 to 0.77), presentation in Cape Town versus Durban (RR 0.76, 95% CI 0.69 to 0.83), concurrent ‘ideal TB management’ (table 1) (RR 0.82, 95% CI 0.75 to 0.89) and querying >2 TB symptoms (from the following four standard screening questions: duration of cough, presence of fever, night sweats, loss of weight or appetite)^22^ (RR 0.90, 95% CI 0.83 to 0.99). GP-related characteristics such as sex, years in practice (≤25 vs >25), country of training (South Africa vs international) or minutes spent with SP (≤10 vs >10) were not associated with antibiotic use or non-use. In a subanalysis on factors contributing to fluoroquinolone use specifically (among GPs who prescribed any antibiotic), only study site was significantly associated (RR of Cape Town vs Durban=0.30, 95% CI 0.14 to 0.64; online supplemental 3).

### Amount paid by SP

The median fee paid by the SP to the provider was US$23/ZAR330 (range=US$0–US$37/ZAR0–ZAR580). Most (89%) of GPs had a single fee regardless of whether medicines were dispensed on site or not, and there was no association between a GP having a two-tiered fee (one price for a consultation; another for consultation +medicines) and the likelihood of a medicine being dispensed (p=0.646). In 12 interactions involving 11 GPs, no fee was charged, and no medicines were directly dispensed. Among all 511 interactions, the average amount paid was US$4/ZAR67 higher when a medicine was dispensed (p=0.000), and the amount paid was on average US$0.60/ZAR9 (95% CI US$0.26 to US$0.91, ZAE4–ZAR14) higher for each additional medicine dispensed. The median consultation fee varied by study site (Durban=US$20/ZAR305 vs Cape Town=US$2/ZAR333, p<0.001).

## Discussion

This study adds to a growing body of work on antibiotic use and prescribing practices in response to clinical presentations of presumptive or confirmed TB in South Africa’s private sector.^23 24^ The study drew on SP methodology, which uniquely assesses real life practices, rather than provider’s knowledge or intended practices. The study illuminates how initial management of people presenting to the private sector with TB symptoms could be associated with the inappropriate use of antibiotics and compromise the timely diagnosis of TB. We discuss the implications of the study findings on quality of TB care, antimicrobial stewardship and health expenditures. We also suggest opportunities for engaging private GPs, who remain a crucial entry point into the health system, to bridge the public–private divide and strengthen responses to TB, HIV-associated TB, as well as medicine and antibiotic use more broadly.

GPs offered medicines in nearly 90% of interactions, and the vast majority were directly dispensed—facilitating potential immediate use—rather than prescribed. Medicine overuse carries the risk of adverse drug events,^25^ and injections, that were offered in nearly a third of interactions, carry additional risks of blood borne infections.^26^ GPs in this study were all registered with HPCSA the regulatory authority that oversees registration and practising of health professionals to ensures patient safety. The common use of medicines may be explained in part by perceived patient expectations and norms about medical service provision, that have been described elsewhere.^27–31^ Injections, for example, are perceived in some settings to have greater efficacy and potency than oral remedies.^32–34^ These perceptions, and GPs’ response to them, may also be heightened in the client- and business-centred environment of private practice. Interactions were on average 8.1 min, and interactions that resulted in medicines tended to be shorter than those that did not, although this relationship was not significant (6.7 vs 8.2 min, p=0.056). It is possible that GPs who were unable to devote adequate time to counsel on non-pharmaceutical approaches to symptom management were more easily able to resort to pharmaceutical options.^29^ As prescribing practices varied significantly by study site, other sociocultural, geographical and demographic drivers may have also been at play. This may include different systems of referral between public and private sectors, differential access to POC testing, or even different patient expectations based on previous experiences with the healthcare system.

Among the full range of medicines provided, antibiotics were represented in over 75% of interactions. This substantiated the results of recent systematic reviews describing high rates of antibiotic prescription in other low-income and middle-income countries including India, China and Kenya.^8 9^ In this study, most antibiotics (86%) were from the ‘Access’ category, with lower potential of selecting for resistance. Only 12% of antibiotics were from the ‘Watch’ category, compared with higher rates observed in similar studies (47.6% in India,^9^ 16% in Ecuador^35^ and Ethiopia,^36^ 78.4% in China,^37^ and no antibiotics were dispensed from the ‘Reserve’ category. The proportion of ‘Access’-group antibiotics fell within the range recommended by WHO (ie, at least 60% of all antibiotics prescribed), thus resulting in a more limited potential for resistance selection. Yet, the overall antibiotic use was still elevated and markedly higher than its use for similar standardised case presentations of TB in the South African public sector (8%).^10^ Most notably, ‘Watch’ antibiotics were more commonly used than in the public sector (12% vs 5%).^38^ Of these ‘Watch’ antibiotics, 68% overall (and 63% of those dispensed to presentations of confirmed TB, ie, case 2) were fluoroquinolones. Although fluoroquinolone use (7.7%) was lower than that observed in other settings such as India (18%),^39^ it was higher than in the South African public sector (0%)^10^ and, consistent with general medicine prescribing practices, higher in the city of Durban (12%) than Cape Town (3%).

These findings have serious potential implications for quality of care in TB and antimicrobial stewardship, that is, optimising antibiotic use to mitigate antimicrobial resistance and infectious disease transmission, and protect patient safety.^40 41^ Prescription of antibiotics prior to initiating a TB test, and especially prescription of fluoroquinolones, carries the risk of delaying TB diagnoses by masking symptoms and thus delaying test taking and/or diagnostic confirmation.^2–5 42 43^ Inappropriate use of fluoroquinolones is further associated with fluoroquinolone-resistance.^44^ ^45 46^ There is thus room for building antimicrobial stewardship, now considered integral to health systems strengthening, and to bridging gaps between patient safety and quality of care in primary health settings in the private sector.^40 41^ However, the use of antibiotics is fraught with complexity. Reports of overuse are balanced by underuse, including in people living with HIV (PLHIV),^47 48^ for whom antibiotics are a vital line of defence against opportunistic infections.^49 50^ In this study, antibiotics were more commonly used when the standardised case history included a known diagnosis of HIV infection (cases 1 and 3), as was co-trimoxazole more specifically, which is recommended as prophylaxis against opportunistic infections among PLHIV. Postinteraction knowledge surveys conducted alongside our primary research^16^ showed that GPs often considered TB in the differential diagnosis (indeed, TB was mentioned in some capacity by the GP in over 80% of visits), but weighed it against other common diagnoses with similar clinical presentations, including community-acquired pneumonia, acute bronchitis, or acute exacerbations of asthma or chronic obstructive pulmonary disease (see online supplemental 4 for a clinical comparison of differentials of SP case presentations).

In interactions where SPs were asked to return for follow-up, they were also more likely to receive an antibiotic, suggesting empiric antibiotic therapy may have been used as a diagnostic tool. This is common practice in TB despite the low sensitivity (67%) and specificity (73%) for diagnosing pulmonary TB, which is well below international standards.^51 52^ On the other hand, in interactions in which >2 TB symptoms were queried, the SP was referred for a TB test and/or to the public sector, or the SP produced a laboratory report indicating GeneXpert-confirmed TB (case 2), antibiotics were significantly less likely to be prescribed. This validates the importance of comprehensive history-taking, and appropriate diagnostic testing in stewarding the appropriate use of therapeutics for respiratory infections. While the South African Standard Treatment Guidelines (2018) recommend empiric antibiotic therapy for other presumed respiratory infections such as pneumonia, they recommend concurrent testing of sputum by GeneXpert to exclude a diagnosis of TB.^19 20^ Enabling use of or access to rapid POC testing such as digital chest X-rays or GeneXpert could mitigate antibiotic misuse and support best practices for TB in initial clinical encounters. However, these tests are also cost prohibitive for small GP practices and cash-paying patients who may not have medical insurance. Evidence from one study suggests the South African public sector initiates TB tests for presentations of basic TB-like symptoms at nearly twice the rate of the private sector (81% vs 43%).^10^ Hence, there may be value in strengthening partnerships between both sectors as has been recently identified by South Africa’s National TB Programme and is envisaged in the National Health Insurance scheme; such programmes are however currently still limited. Other sets of providers can also be engaged in the process of antimicrobial stewardship, including pharmacists and microbiologists.^53^ Innovative investments enabling the utilisation of high-quality POC diagnostics in private health facilities have been successfully implemented in other settings such as India.^54 55^

Overuse of medicines also has financial implications. In this study, the amount paid by the SP was significantly higher when medicines were dispensed (table 2), and over 60% of medicines were not part of the South African EML that is designed to support use of the most efficacious and most cost-effective medicine choices.^20^ Although increases in patient costs attributable to medications were marginal (US$4/ZAR67), and medication dispensation may have been connected to perceptions around quality of care and patient demand, observed prescribing practices may still be contributing to increases in healthcare expenditure. The private sector accounts for 84% of all pharmaceutical spending (roughly ZAR33.2 billion/US$.9 billion annually), and practices that facilitate use of non-EML medicines could contribute to these growing costs.^56^ Further research is needed to better understand and manage patient expectations, the limitations in which GPs practice, and to strengthen stewardship measures in the private sector to support GP-patient communication, reduce medicine overuse and improve adherence to EML guidance.

This study had several limitations. First, to minimise SP detection, we observed single healthcare encounters and were unable to evaluate potentially complementary (or harmful) practices within follow-up visits. Second, despite rigorous SP training, the novel practices, accents and medical terminology used in real-life interactions may have contributed to recall bias. Thirdly, while SP methods overcome performance and Hawthorne biases common in observational, survey-based or vignette-based quality of care studies, the fact that SPs were actors and did not have overt symptoms of TB on physical examination (eg, fever, wasting, abnormal breath sounds) may have biased GPs away from a diagnosis of TB. However, TB can commonly present without physical symptoms, especially in immunocompromised patients, and thus should remain high on the differential diagnosis (particularly in communities with high rates of TB). Fourthly, although we suggest GPs’ practices may have been driven by perceptual and social factors, we did not directly inquire on their rationale or intended duration of recommended prescriptions. This could help to distinguish appropriate medicine use from misuse. For example, it was not possible to distinguish if sulfamethoxazole/trimethoprim (ie, co-trimoxazole preventive therapy) was prescribed to prevent opportunistic infections in SPs who disclosed a positive HIV status (cases 1 and 3) in accordance with national HIV management guidelines, versus used as a broad-spectrum antibiotic to treat an unknown respiratory infection. Finally, our study was conducted prior to the global pandemic of COVID-19 and thus may not represent what would be observed today; in fact, we expect that the challenges we highlighted may be further exacerbated given the similar symptom profile between COVID-19 and TB.^57 58^ Similarly, these results and our interpretations may not be generalisable to contexts outside of our high-TB burden, low-middle-income study sites.

## Conclusion

This research contributes novel insights into prescribing practices of GPs when presented with symptoms suggestive of TB and alerts us to the potential for these practices to drive health system expenditures, patterns of antibiotic use and TB diagnostic delay. The strategy of ‘treatment as diagnosis’ may be spurred by diagnostic uncertainty, inaccessibility to POC tests in private facilities, and patient demand for medicines. GPs are, however, key players shaping patients’ pathways in the health system. Further inquiry into their practices and engaging them in gatekeeping and stewardship measures are likely to have widescale impacts in timely and appropriate management of TB, HIV and related infections.

## Data Availability

All data relevant to the study are included in the article or uploaded as online supplemental information.
