# Supplementary material for: Prescribing practices for presumptive TB among private general practitioners in South Africa: a cross-sectional, standardised patient study
Source: BMJ Glob Health. 2022 Jan 18;7(1):e007456. doi: 10.1136/bmjgh-2021-007456 (PMC8768922; doi:10.1136/bmjgh-2021-007456)
Supplement: Supplementary data [file bmjgh-2021-007456supp001.pdf]

Prescribing practices for presumptive TB among private general practitioners in South Africa: Online Supplementary

Supplemental 1. Consent diagram

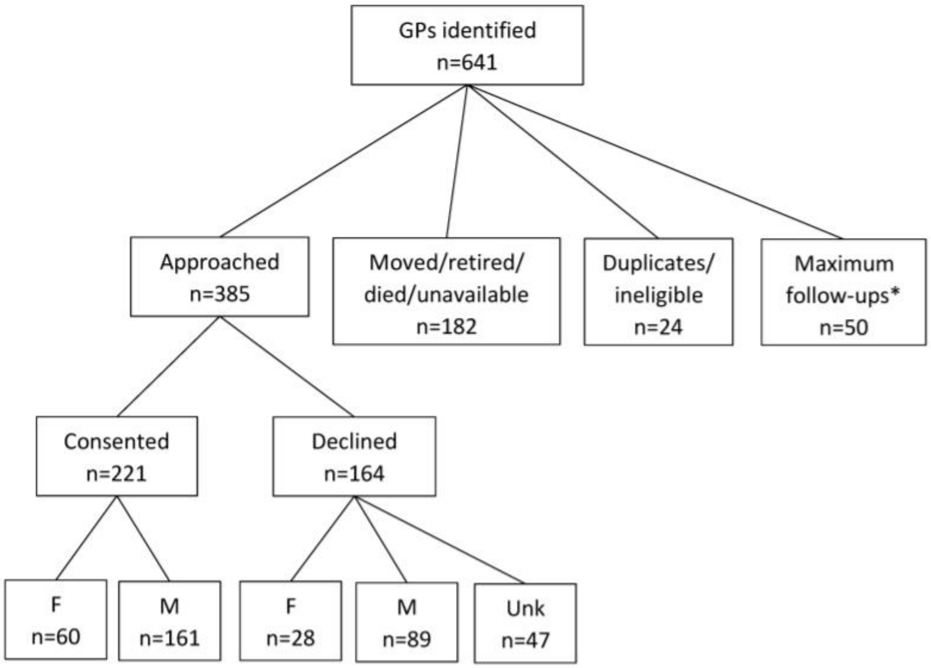

*GPs were approached a maximum of 5 times by recruitment staff
Abbreviations: GPs=general practitioners, Unk=unknown

Supplemental 2. All active ingredients and concordance with South African Essential Medicines List

Active Ingredient	Frequency	Percentage	Classification	SA EML
Theophylline	416	11.94	bronchodilator	no
Paracetamol	372	10.67	analgesic/ anti-pyretic	yes
Ammonium Chloride	296	8.49	cough remedy	no
Diphenhydramine	295	8.46	cough remedy	no
Sodium Citrate	278	7.98	cough remedy	no
Amoxicillin	276	7.92	antibiotic	yes
Caffeine	219	6.28	other	no
Chlorphenamine	195	5.6	antihistamine	yes
Phenylephrine	144	4.13	decongestant	no
Codeine Phosphate	132	3.79	analgesic/ anti-pyretic	no
Ascorbic Acid	111	3.19	vitamin	no
Prednisone	91	2.61	steroid	yes
Meprobamate	60	1.72	antidepressant/anxiolytic	no
Sulfamethoxazole	59	1.69	antibiotic	yes
Trimethoprim	59	1.69	antibiotic	yes
Clavulanic Acid	51	1.46	antibiotic	yes
Multivitamin	42	1.21	vitamin	yes
Vitamin B	40	1.15	vitamin	yes
Ciprofloxacin	35	1	antibiotic	yes
Ibuprofen	26	0.75	NSAID	yes
Doxycycline	23	0.66	antibiotic	yes
Ephedrine	18	0.52	decongestant	no
Cetirizine	16	0.46	antihistamine	yes
Loratidine	15	0.43	antihistamine	no
Pseudoephedrine	15	0.43	decongestant	no
Diclofenac	14	0.4	NSAID	no
Erythromycin	13	0.37	antibiotic	no
Triprolidine	13	0.37	antihistamine	no
Salbutamol	10	0.29	bronchodilator	yes
Guaiphenesin	9	0.26	cough remedy	no
Herbal	9	0.26	herbal	no
Dextromethorphan	8	0.23	cough remedy	no
Doxylamine Succinate	8	0.23	antihistamine	no
Cefalexin	7	0.2	antibiotic	no
Bromhexine	6	0.17	cough remedy	no
Metronidazole	6	0.17	antibiotic	yes
Orciprenaline	6	0.17	bronchodilator	no
Levocetirizine	5	0.14	antihistamine	no
Phenylpropanolamine	5	0.14	decongestant	no

Naproxen	4	0.11	NSAID	no
Beclomethasone	3	0.09	steroid	no
Azithromycin	3	0.09	antibiotic	yes
Isoniazid	3	0.09	antibiotic	yes
Etofylline	3	0.09	bronchodilator	no
Indometacin	3	0.09	NSAID	no
Mefenamic Acid	3	0.09	NSAID	no
Meloxicam	3	0.09	NSAID	no
Mepyramine	3	0.09	antihistamine	no
Oxytetracycline	3	0.09	antibiotic	no
Pholcodine	3	0.09	cough remedy	no
Cefuroxime	2	0.06	antibiotic	no
Atenolol	2	0.06	beta-blocker	yes
Ethambutol	2	0.06	antibiotic	yes
Fluticasone	2	0.06	steroid	yes
Iron	2	0.06	vitamin	yes
Orphenadrine Chloride	2	0.06	other	yes
Diphenpyraline	2	0.06	antihistamine	no
Pryazidamine	2	0.06	antibiotic	yes
Rifampicin	2	0.06	antibiotic	yes
Levofloxacin	2	0.06	antibiotic	no
Oxycodone	2	0.06	analgesic/ anti-pyretic	no
Promethazine	2	0.06	antihistamine	no
Aceclofenac	1	0.03	NSAID	no
Acetylsalicylic Acid	1	0.03	NSAID	no
Acyclovir	1	0.03	anti-viral	yes
Amitriptyline	1	0.03	antidepressant/anxiolytic	yes
Betamethasone	1	0.03	steroid	yes
Budesonide	1	0.03	steroid	yes
Benzocaine	1	0.03	other	no
Diazepam	1	0.03	antidepressant/anxiolytic	yes
Flucloxacillin	1	0.03	antibiotic	yes
Folic Acid	1	0.03	vitamin	yes
Methyl Salicylate	1	0.03	NSAID	yes
Metoclopramide Hcl	1	0.03	other	yes
Cefadroxil	1	0.03	antibiotic	no
Moxifloxacin	1	0.03	antibiotic	yes
Cetylpyridinum	1	0.03	other	no
Salicylamide	1	0.03	NSAID	yes
Codeine	1	0.03	analgesic/ anti-pyretic	no
Docusate Sodium	1	0.03	other	no
Hydrobromide	1	0.03	cough remedy	no
Montelukast	1	0.03	bronchodilator	no

Panthenol	1	0.03	other	no
Pheniramine	1	0.03	antihistamine	no
Probiotic	1	0.03	vitamin	no
Thiocolchicoside	1	0.03	other	no
**Total Active Ingredients**	3485			

Supplemental figure 3. Factors associated with fluoroquinolone use

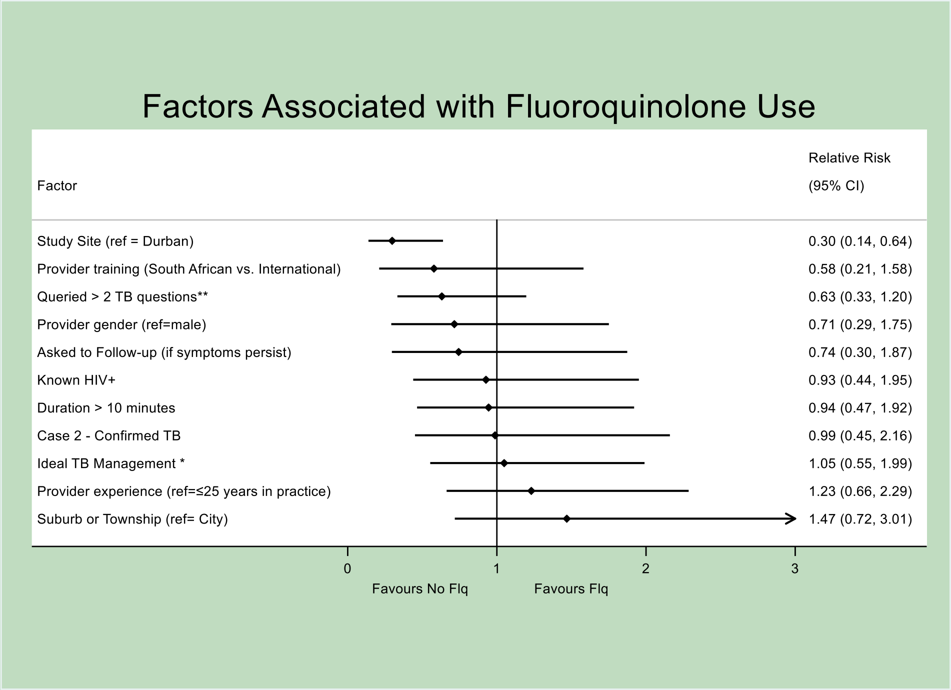

Supplemental figure 4. Differential diagnoses of SP Case presentation

**Condition**	**Tuberculosis**[7]	**Community-Acquired Pneumonia (CAP)**[55]	**Acute Exacerbation of COPD** [56]	**Acute bronchitis**[31]
**Aetiology**	*M. tuberculosis*	- *S. pneumoniae*<br>- *S. aureus*<br>- *H. influenzae*<br>- PLHIV*: P jiroveci, M. Tuberculosis*	**Viral** (up to 50%)<br>**Bacterial**<br>- *H. influenzae*<br>- *S. pneumoniae*	**Viral** (90%)<br>**Bacterial**<br>- *M. pneumonia*<br>- *C. pneumonia*<br>- *B. pertussis*
**Clinical Features**	Subacute or chronic symptoms >2 weeks (cough with sputum, hemoptysis, fever, loss of weight/ appetite, night sweats)	- Acute symptoms <2 weeks (cough +/- sputum, fever, pleuritic chest pain, dyspnea)<br>- Abnormal vital signs: fever, tachycardia, tachypnea<br>- Consolidation on lung auscultation	- Acute increase in baseline dyspnea, cough and/or sputum above normal variation, requiring change in COPD medication<br>- Tachycardia, tachypnea	- Initially, non-productive cough that may or may not become productive<br>- Fever<br>- +/- viral URTI symptoms
**Diagnosis**	Xpert MTB/RIF, sputum smear or culture	- Clinical diagnosis **may** be sufficient<br>- Definitive = CXR (new or worsening consolidation)	- Previous diagnosis of COPD (using spirometry) + above clinical findings	- Exclusion of other LRTI
**Management**	- RHZE x 2 months (initial), RH x 4 months (continuation)	- **Uncomplicated**: oral amoxicillin<br>- **PLHIV**: oral amoxicillin-clavulanate acid OR moxifloxacin/ levofloxacin	- Short-acting inhaled B_2 agonists (SABA), short-course oral corticosteroids, +/- antibiotics (if evidence of severe exacerbation)	- **Uncomplicated**: supportive (self-limiting)<br>- Antibiotics not indicated in absence of COPD
